# Results of a multicenter, randomized trial examining a new transition model for post-kidney transplant adolescents

**DOI:** 10.1038/s41598-025-95845-7

**Published:** 2025-04-03

**Authors:** Martin Kreuzer, Jenny Prüfe, Marie-Luise Dierks, Silvia Müther, Dirk Bethe, Anja Büscher, Krisztina Heindl-Rusai, Sabine Hollenbach, Bernd Hoppe, Ulrike John-Kroegel, Nele Kirsten Kanzelmeyer, Günter Klaus, Birgitta Kranz, Jun Oh, Martin Pohl, Susanne Rieger, Bettina Ruckenbrodt, Katja Sauerstein, Hagen Staude, Christina Taylan, Julia Thumfart, Marcus Weitz, Rieke Ringlstetter, Anika Großhennig, Lars Pape

**Affiliations:** 1 Clinic of Pediatrics II, Essen University Hospital, Hufelandstrasse 55, 45147 Essen, Germany; 2 Department of Epidemiology, Social Medicine and Health System Research, Hannover Medical School, Carl-Neuberg-Strasse 1, 30625 Hannover, Germany; 3 Berliner TransitionsProgramm (BTP), DRK-Kliniken Berlin Westend, Spandauer Damm 130, 14050 Berlin, Germany; 4 Division of Pediatric Nephrology, Center for Child and Adolescent Medicine, Heidelberg University Hospital, Im Neuenheimer Feld 672, 69120 Heidelberg, Germany; 5 University Children’s Hospital, Währinger Gürtel 18-20, Vienna, 1090 Austria; 6 KfH Center of Pediatric Nephrology, St. Georg Hospital, Delitzscher Str. 141, 04129 Leipzig, Germany; 7 Bonn Center of Pediatric Nephrology, Im Mühlenbach 2b, 53127 Bonn, Germany; 8 Pediatric Nephrology, Universitätsklinikum Jena, Kastanienstraße 1, 07747 Jena, Germany; 9 Department of Pediatric Kidney, Liver and Metabolic Diseases, Hannover Medical School, Carl- Neuberg-Strasse 1, 30625 Hannover, Germany; 10 KfH Center of Pediatric Nephrology, University Hospital of Marburg, Baldingerstraße, 35043 Marburg, Germany; 11 University Children’s Hospital Münster, Albert-Schweitzer-Campus 1, 48149 Munster, Germany; 12 University Children’s Hospital Eppendorf, Martinistraße 52, 20251 Hamburg, Germany; 13 Department of General Pediatrics, Adolescent Medicine and Neonatology, Freiburg University Hospital, Heiliggeiststraße 1, 79106 Freiburg im Breisgau, Germany; 14 Children‘s Hospital, Olgahospital Klinikum Stuttgart, Kriegsbergstraße 60, 70174 Stuttgart, Germany; 15 Children’s Hospital, University of Erlangen, Maximiliansplatz 2, 91054 Erlangen, Germany; 16 University Children’s Hospital, Ernst-Heydemann-Straße 8, 18057 Rostock, Germany; 17 Pediatric Nephrology, University Hospital of Cologne, Kerpener Str. 62, 50937 Cologne, Germany; 18 Department of Pediatric Nephrology, Campus Charité Virchow Klinikum, Augustenburger Platz 1, 13353 Berlin, Germany; 19 University Children’s Hospital Tübingen, Geissweg 3, 72076 Tübingen, Germany; 20 Institute of Biostatistics, Hannover Medical School, Carl-Neuberg-Strasse 1, 30625 Hannover, Germany

**Keywords:** Kidney, Renal replacement therapy, Paediatrics

## Abstract

Allograft loss after pediatric kidney transplantation (KTx) is highest in adolescents and young adults. Non-adherence and Health Care Transition (HCT) are important factors, but others also contribute. In the TransNephro study patients were randomized 1:1. The intervention group was included in the Berlin Transition Program (BTP) and incorporated a central case manager, a communication app, and joined transition rounds for one year before and one year after transfer. Primary endpoint was the coefficient of variation (CoV) of the trough level of the calcineurin inhibitor as a surrogate marker for medication adherence associated with graft loss. Least square (LS) mean differences and corresponding 95% confidence intervals (CIs) were estimated using an analysis of covariance (ANCOVA) model. We assessed 220 patients for eligibility. 49 patients were randomized to the intervention group and 53 to the control group. We analyzed 84 patients in the modified intention-to-treat analysis (38 intervention, 46 controls) and 60 in the per protocol analysis (25 intervention, 35 controls). We found no difference in CoV. We saw low numbers of graft-related events and observed no differences with respect to quality of life. BTP did not improve adherence and other outcome parameters. Non-adherent patients may have decided not to participate, whilst adherence of participants was already good at study start. It is therefore achallenge to design future multicenter trials on HCT that include multiple interventions.

Trial registration: ISRCTN22988897, 24/04/2014, 10.1186/ISRCTN22988897.

**Supplementary Information:**

The online version contains supplementary material available at 10.1038/s41598-025-95845-7.

## Introduction

Transition of chronically ill adolescents from pediatric to adult medicine (Health Care Transition, HCT) should at least conserve their state of health^1^. The transition process can be considered successful if it promotes the patient’s health competence, supports their psychosocial rehabilitation, and improves their self-determination efficacy, including their ability to make decisions and communicate about their care^2^. However, up to 40% of adolescent patients lose access to special care during the HCT from pediatric to adult services^3^. In nephrology, in particular, transition is a significant point in patient care^1,3^. Research shows that more kidney allografts are lost in patients aged between 16 and 21 years than in any other age group^4,5^. Graft failure requires a return to compulsory dialysis, which reduces quality of life (QoL) and leads to considerable additional healthcare costs. Decreasing adherence in adolescence is a major factor for a reductionin graft function and graft loss in this age group. Adolescents often struggle with compliance because they want to be independent, and because of peer influence, forgetfulness, and an underdeveloped sense of long-term consequences. Emotional variability, complex treatment regimens, and misinformation further contribute^2–4^. Moreover, preterm graft losses increase mortality and shorten life expectancy.

Increase of health-related quality of life (HRQoL) is also an important goal of kidney transplantation (KTx) and an additional significant factor in the transition process^3^. In addition, it has been proven that HRQoL is a very important factor in the future employment of a young adult^21^.

To avoid negative consequences for both the individual and society, a structured and planned HCT process is necessary. Several national and international recommendations and guidelines on HCT have been published^2,3,6^, but most of these are based on expert opinion rather than research data. This is simply because of the scarcity of randomized controlled data on HCT. Our current literature research revealed only a few randomized controlled trials within the past 10 years covering congenital heart disease and diabetes type 1^7,8^. Nonetheless, several studies on the impact of transition strategies have been published within the past two decades^9,10^. Structured transition interventions often resulted in positive outcomes. Workshops, joint visits, or multidisciplinary appointments may be particularly effective components.

The Berlin Transitions Program(BTP) is the first structured transition program in Germany to be financed by statutory health insurance^1^. It manages the HCT of adolescents and young adults (AYA) with different chronic conditions (e.g., diabetes mellitus type 1, epilepsy, renal diseases, juvenile rheumatoid arthritis, inflammatory bowel diseases, and neuromuscular diseases) from pediatric to adult care over a period of two years. It contains specific transitional measures for in-depth communication between the pre- and post-transfer treating physicians, as well as conversations with adolescent patients and their parents, structured in terms of time and content. A case management protocol is used to support and ensure the unhindered flow of information and the application of treatment measures.

The introduction of smartphone apps, which can support adolescent patients, may improve their adherence^11^. The rationale for using such apps is to interact with the patients through media that are familiar to them, enabling them to manage their disease even under the challenging conditions of adolescence. A willing acceptance of high-tech tools by adolescent patients can support and develop their competency in dealing with the disease.

We hypothesized that a structured transition, in this case the BT, complemented by telemedicine support via smartphone apps, would improve the therapeutic adherence of adolescent (KTx recipients. If correct, this approach would improve post-transfer functioning and KTx survival, and ultimately patient survival and wellbeing. We have chosen the BTP for this study as it is the only transition program in Germany that is funded by public health insurance, and so could thus be implemented, in routine care.

We conducted a prospective, multicenter, two-arm randomized intervention study to clarify whether a structured transition program with supplementary smartphone apps can improve adherence during HCT in AYA KTx patients.

## Methods

Consort reporting guidelines were applied^12^.

### Study design

For more detailed information, refer to the published study protocol^1^, but in brief, this was a prospective, multicenter, randomized two-arm intervention study. In the control group, adolescents went through the HCT protocol routinely used at their own center. In the intervention group, adolescents were transitioned using all structural elements from the BTP, that is, case managers and transition interviews and a smartphone (Samsung Galaxy Y, Samsung, Seoul, South Korea or own) with two apps. The duration of the overall intervention was two years. Transition interviews were performed one year before transfer, at time of transfer, and one year after transfer based on structured questionnaires. Detailed information (in German) can be found at http://www.btp-ev.de. One app developed by the BTP (“WhatsApp style”) was used to communicate with the participants, who were contacted monthly by the case manager and asked if they needed support; this was consistently applied for all participants. The patients could use the app to pose any other questions to the case manager, who replied within 24 h during working hours. The other app provided a medication plan, including a reminder function, and a diary for notes and blood pressure data. This was a standard medication plan app. At study start the apps were explained to the patients by the local study nurses and the initial medication plan was entered in the app collaboratively by study nurse and patient. A cell phone and a data plan were provided if needed. The same case manager took care of all the patients. The patients were included as routine BTP patients and BTP procedures were not modified.

Patients were randomized between October 2014 and February 2019 at 22 study sites across Germany and Austria. Cooperating adult nephrologists were informed about this trial by the German Society of Nephrology and asked if they were willing to participate after transfer.

The study protocol, the patient consent form, and the amendment (in which a third stratum for non-autonomous patients was introduced) was approved by the leading research ethics committee at Hannover Medical School (approval number: 6660) and by local ethics committees of the other 22 participating centers. All methods were performed in accordance with the relevant guidelines and regulations including the Declaration of Helsinki.

### Patients

Recruitment was conducted by the pediatric nephrologist in every center together with the psychosocial teams. Patients 1 year before planned transfer were contacted during a routine outpatient visit and written information about the study was given to them. At the next routine visit, study participation was discussed with the patients and their guardians. At a study meeting before study start, the local investigators were informed about the recruitment process in order ensure harmonization between the centers. Informed consent was obtained from the adolescent patients, as well as from their parents/ guardians. The study cohort included male and female KTx patients between 16 and 22 years of age at study enrollment from 17 different study sites. Included were participants with at least 14 months to expected transfer to adult medical care, at least three months post-transplantation, capable of operating a smartphone, and able to provide informed consent. Exclusion criteria were signs of acute transplant rejection or indications of a need for dialysis in the near future (since transplant survival was a study outcome variable), as well as mental incapacity (since this could prevent complete and independent use of the apps). Though patients with mental incapacity were not randomized in the study, they could be considered on a purely observational basis (amendment 1). An on-site physician involved in the study obtained consent from patients who met the inclusion criteria, and also from parents/ guardians of patients under the age of 18 years. Identification of eligible patients began in the first year of the study. Addresses of patients and physicians were registered so that contact could be maintained, in case of a change in care providers.

### Randomization

Randomization was stratified by the three most important prognostic factors: time since KTx (< 1 year, 1–5 years, > 5 years), gender (male, female) and site. Although the stratification of the randomization by study site led to a high number of overall strata, the stratification for center would ensure that for centers with and without experience in transition patients were enrolled randomly to both groups and that the different approaches used in adult centers after transition were distributed equally in both groups. To reduce the number of potential broken blocks, a block randomization with a variable block length was used for the generation of the randomization lists.

### Procedures

The study timeline began with the start of transition and concomitant entry (E) into the study at time point T0 (baseline), and proceeded with a 12 (to 14)-month assessment at time point T1 and a 24 (to 26)-month assessment at time point T2.

Baseline data were documented once retrospectively. The course of calcineurin inhibitor levels and estimated glomerular filtration rate (eGFR), the number of acute rejections (examined every 4–8 weeks), and the detection of donor-specific antibodies, if assessed, were recorded as surrogate parameters of therapy adherence. This diagnostic schedule was in accordance with standard German healthcare and would be followed regardless of participation in the study. No blood samples were collected beyond those collected throughout the routine course of care.

In the control group, transition was performed according to local standards. In Germany and Austria most centers had no detailedtransition programs but some local procedures for transfer had been implemented.

The patients took their medications as usual when under routine care; however, we did not assess whether the caregiver supervised this.

### Choice of primary endpoint measure

The long-term goal of this trial was to improve adherence through an assisted and controlled transition process. The primary outcome variable of the study was therapy adherence, as indicated by individual variability in calcineurin inhibitor trough levels. Trough levels were recorded at all regular follow-up examinations (every 4–12 weeks). A coefficient of variation (CoV, in %) was calculated for each patient based on these examination data with a minimum of three measurements. These coefficient values served as surrogate parameters for patient adherence. The individual CoV for immunosuppressive agent levels is the endpoint of choice for this field of research and several publications consider the best surrogate parameter for adherence^1,13,14^. It has also been proven that there is a significant correlation between CoV and graft failure as well as with the risk of antibody-mediated rejection^15^.

### Secondary endpoints

The secondary endpoints of the study were eGFR (using the Schwartz 2009 formula^16^) and serum creatinine levels. In addition, we documented transplant-related outcomes such as allograft and patient survival, as well as acute rejection reactions (presence of donor-specific antibodies and chronic-humoral rejection if available). Patient-reported outcome measures were ascertained using standardized questionnaires and interviews. Specifically, SF-12 (HRQoL-short form^17^)and FSozU (questionnaire on social support^18^) were assessed once 6 months after transfer in the adult medicine phase of the study in paper-pencil format. The questionnaire was sent by mail to the families and included a stamped envelope for return.

### Statistical methods

Derivation of sample size is extensively described in Kreuzer et al.^1^. Briefly, we expected a variation coefficient of 0.26 in the control group compared to 0.18 in the intervention group and a common standard deviation of 0.15. Based on these assumptions, a two-sided type-I error of 5% and a power of 80%, with a Student’s two-sample t-test, a sample size of 57 patients per group was calculated and a sample size of 114 patients was initially planned for recruitment.

The primary analysis was conducted on the modified intention-to-treat (mITT) population including all patients that had been randomized. The primary endpoint CoV of the trough level of the calcineurin inhibitor was analyzed using an analysis of covariance (ANCOVA) including the treatment group (intervention/control), randomization stratum variable (for time since Tx and gender, see above) and study site in the ANCOVA model for adjustment. Least square (LS) means differences for the difference between intervention and control group and respective 95% confidence intervals (CIs) were estimated. A negative LS means difference for the primary endpoint indicated a favorable outcome for the intervention group. According to the pre-specification in the study protocol, CoV of patients with fewer than three available trough level measurements were replaced by the largest CoV in the respective treatment group. The CoV of patients with graft loss or the necessity of dialysis only included trough level measurements before the respective event.

In addition, we performed extensive sensitivity analyses. Specifically, we performed ANCOVA without adjustment only for the treatment group, adjustment only for the treatment group and the randomization stratum. Furthermore, we repeated all analyses in the per-protocol (PP) population, which comprised all patients with an available CoV value after transition.

Mean eGFR at the adult clinic and mental and physical subscales of the SF-12 were analyzed in line with the primary analysis and adjusted for the respective baseline values using ANCOVA models in all patients with available data. Sensitivity analyses without adjustment and adjustment only for the randomization stratum variable for the secondary endpoints were also performed in the ITT and PP populations. Transplant-related secondary outcomes are reported in terms of absolute frequencies. All analyses were performed using SAS 9.4. The trial has been registered at clinicaltrials.gov (number: ISRCTN22988897, date of registration: April 25, 2014; last update:14.06.2017, Web-link: https://www.isrctn.com/ISRCTN22988897).

### Role of the funding source

The TRANSNephro study was funded by the KfH (Kuratorium für Dialyse und Nierentransplantation) Stiftung Präventivmedizin. The funder of the study had no role in the study design, data ascertainment, analysis, interpretation, and writing of the manuscript.

## Results

### Study population and baseline characteristics

One Austrian and 21 German pediatric KTx centers agreed to participate in this study. Although the study was open at 22 sites, data collection only occurred at 17 sites and the recruiting time had to be extended multiple times and lasted from October 2014 to February 2019 equating to 52 months.

**Fig. 1:**
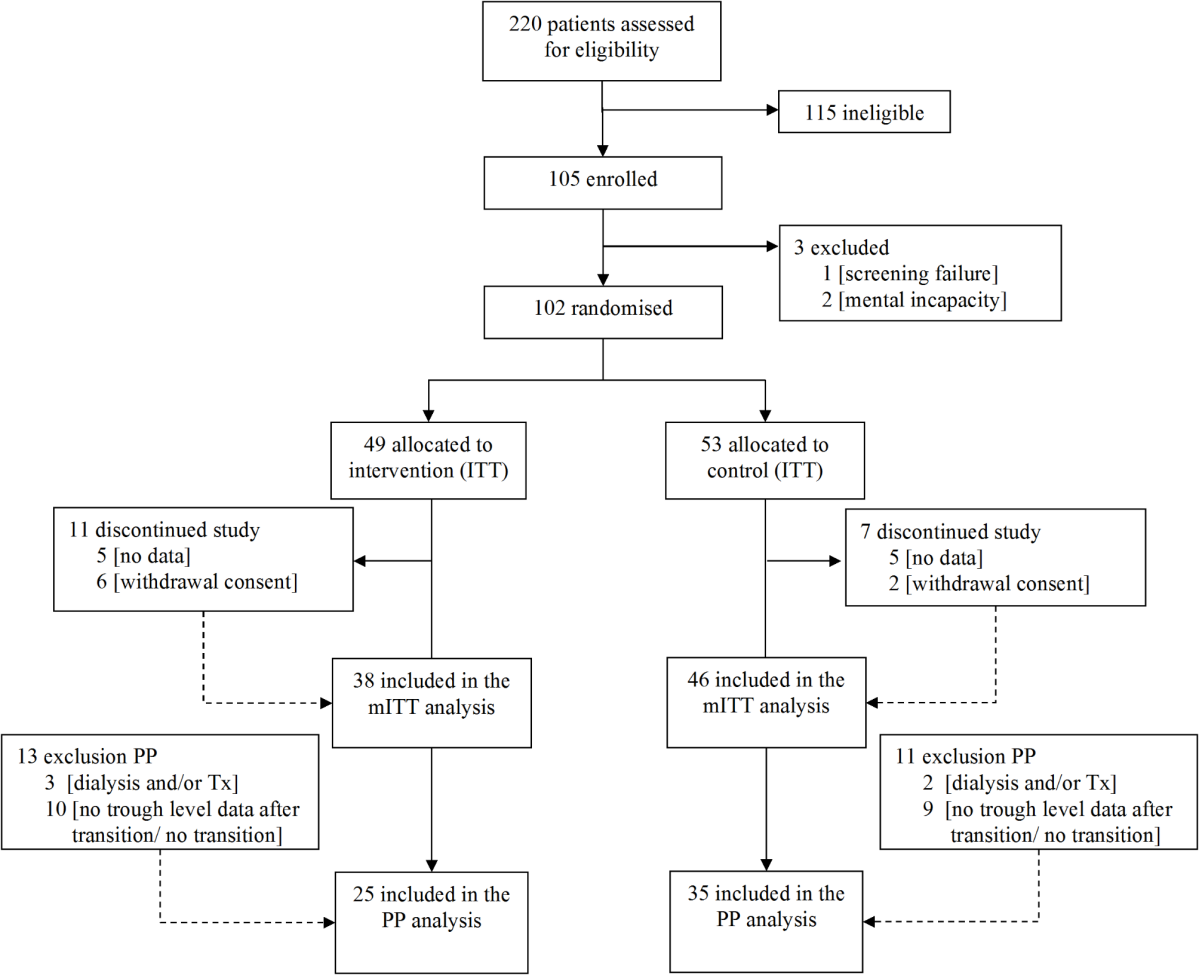
Consort diagram.

**Fig. 2:**
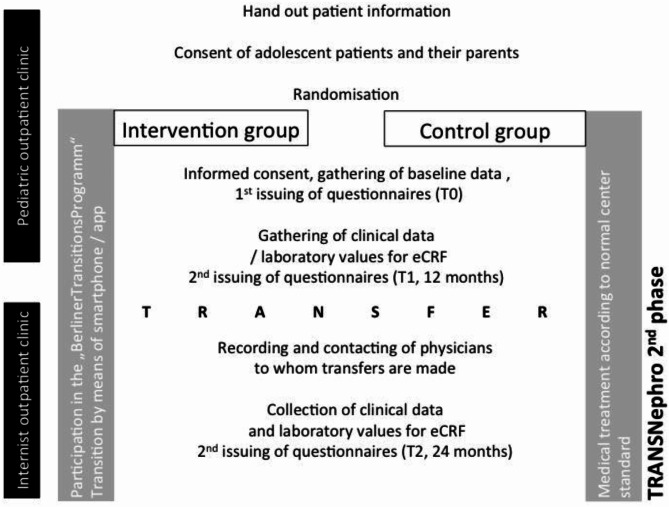
Structure of the intervention study.

A total of 220 patients were assessed for eligibility (see consort diagram, Fig. 1). Of these, 118 were not included in the trial. The majority (96%, *n* = 115) declined participation because they didn’t feel they had need of a time-consuming transition program to improve their care in adult medicine. Finally, 102 patients were randomized: 49 into the intervention and 53 into the control group respectively. Of those randomized participants, 78% (*n* = 38) in the intervention group and 87% (*n* = 46) in the control group had available data before transition. In the remaining patients, their treating physicians postponed the transition until after the study period. Documentation of the follow-up during and after transition as originally planned (Fig. 2) was—unexpectedly—a tremendous challenge. The loss in follow-up after transfer was a serious problem for the study team and the pediatric caregivers: several patients did not make an appointment with the adult nephrologist, who was projected to continue care after transfer. We wrote letters to the patients and attempted to reach the families by phone several times to avoid loss of follow up, but despite these considerable efforts a substantial number of patients were lost to follow up.

In addition, 10 nephrologists did not cooperate, refusingto provide data. Because of this we also have no complete data on adult nephrology appointment ‘no shows’ and no follow up, an indirect indicator of adherence.

Finally, we only received post-transfer data on CoV measurements in 51% (*n* = 25) of participants in the intervention group and 66% (*n* = 35) in the control group, respectively, and in consequence, the PP population was substantially reduced.

Baseline characteristics for mITT and PP populations are reported in Table 1. In the intervention group, there were no essential differences between the ITT, mITT, and PP populations. Time since Tx was slightly different within and between these groups, but this is probably due to the high variability of this parameter. Importantly, we did not see substantial differences in the respective stratum variable.

**Table 1:** Baseline characteristics of patients. *mITT* modified intention to treat population, *PP* per protocol population, *n* sample size. Displayed are absolute and relative numbers and p-Value of two-sided chi-squared-test for gender and Tx random stratum. For continuous parameters mean, standard deviation, median, minimum and maximum values are depicted and compared by two-sided student’s t-test.

	mITT			PP		
	Intervention	Control	p-Value	Intervention	Control	p-Value
n	38	46		25	35	
Gender, male (%)	23 (60.5%)	24 (52.2%)	0.443	15 (60.0%)	18 (51.4%)	0.511
Age, years	17.7 ± 1.7 (17 / 16–23)	17.4 ± 1.4 (17 / 15–22)	0.348	17.7 ± 1.7 (17 / 16–23)	17.4 ± 1.2 (17 / 16–22)	0.467
Height, cm	166.0 ± 12.0 (168 0.5/ 130–185)	166.2 ± 10.2 (167 / 130–186)	0.932	165.2 ± 12.2 (169/ 130–185)	166.8 ± 8.2 (167 / 151–183)	0.550
Time since Tx, years	8.1 ± 4.7 (7.5/ 0–15)	5.7 ± 4.6 (4.5/ 0–17)	0.024	6.8 ± 4.3 (7/ 0–15)	5.2 ± 4.3 (4/ 0–17)	0.164
Randomization stratum						
1 (male, time since Tx < 5 y)	7 (18.4%)	13 (28.3%)	0.205	6 (24.0%)	10 (28.6%)	0.618
2 (male, time since Tx ≥ 5 y)	16 (42.1%)	11 (23.9%)		9 (36.0%)	8 (22.9%)	
3 (female, time since Tx < 5 y)	6 (15.8%)	13 (28.3%)		5 (20.0%)	11 (31.4%)	
4 (female, time since Tx ≥ 5 y)	9 (23.7%)	9 (19.6%)		5 (20.0%)	6 (17.1%)	

### Primary endpoint

In the ANCOVA analysis of the CoV immunosuppressant trough level, we saw no difference between the intervention and the control groups, both in mITT and PP analyses (see Table 2). The LS mean of the CoV immunosuppressant trough levels are 0.29 in the intervention group and 0.31 in the control group, i.e., nearly the same and thus in both groups larger than initially expected for the intervention group. Nevertheless, the standard deviation was more than twice that expected (intervention group: 0.34 (0.33); control group: 0.37 (0.32)). There were no gender-specific differences.

**Table 2:** Results of analysis of variance (ANCOVA) for the primary endpoint (*CoV* coefficient of variation of immunosuppressant trough level) and secondary endpoints (*eGFR* - estimated glomerular filtration rate and the subscales of the *SF-12* Short form-12 questionnaire). ANCOVA models for the primary endpoint are adjusted for randomization stratum (male/female and time since Tx </≥ 5 years) and study site. ANCOVA models for the secondary endpoints are adjusted for randomization stratum, study site, and respective baseline value/mean of baseline values at the children’s hospital phase. Displayed are least squared (LS) means per group and for the difference (intervention-control group) with respective 95% confidence intervals (CI) and p-values. *mITT* modified intention to treat, *PP* per protocol.

	Population	LS mean	LS mean	LS mean difference (95% CI)	p-value
		Intervention	Control		
CoV immunosuppressant trough level	mITT (*n* = 84)	0.29	0.31	− 0.022 [− 0.19, 0.15]	0.7953
	PP (*n* = 60)	0.24	0.30	− 0.058 [− 0.23, 0.11]	0.4898
Mean eGFR at adult hospital phase	mITT (*n* = 61)	47.6	50.8	− 3.197 [− 9.57, 3.18]	0.3172
	PP (*n* = 55)	50.83	56.2	− 5.366 [− 11.59, 0.86]	0.0890
SF-12 physical subscale at adult hospital phase	mITT (*n* = 27)	42.5	42.1	0.425 [− 1.55, 2.40]	0.6493
	PP (*n* = 24)	42.6	42.0	0.651 [− 1.63, 2.93]	0.5424
SF-12 mental subscale at adult hospital phase	mITT (*n* = 27)	42.2	43.4	− 1.198 [− 7.11, 4.71]	0.6684
	PP (*n* = 24)	44.3	44.4	− 0.06 [− 6.43, 6.32]	0.9852

### Secondary endpoints

No patient died during the study. The number of patients that received dialysis and Txdue to graft loss before and after transition was very small (see Table 3). Specifically, in the intervention group, two graft losses occurred before transfer and one graft loss after transfer. In the control group, we observed one graft loss after transfer, all in patients with a GFR < 30 ml/min at study start. All patients moved to dialysis in case of graft loss; none were retransplanted during the observation period.

**Table 3:** Results of transplant-related secondary outcomes. Absolute numbers are shown.

	Intervention group *n* (mITT) = 38	Control group *n* (mITT) = 46
Death	0	0
Dialysis or transplantation due to graft loss before transition	1	1
Dialysis or transplantation due to graft loss after transition	2	1
Rejection before transition	2	2
Rejection after transition	0	2
Chronic transplant glomerulopathy	0	1

We were unable to obtain data on rejections after the transfer. Within the pediatric phase in the intervention group, two borderline, two BANFF Ia, and one chronic rejection were documented as compared to one BANFF Ia, and two chronic rejections in the controls. Data on donor-specific antibodies were too sparse to analyze. Nevertheless, due to difficult documentation post-transfer, we cannot exclude the possibility that more patients underwent Tx, experienced graft loss, or had more rejections than were reported.

We cannot provide data on cystatin C as it was not measured consistently.

Interestingly, we observed a trend for a remarkably lower mean eGFR in the adult clinic outpatient phase in the intervention group compared to controls in both the mITT analysis (LS mean: 47.6 in the intervention vs. 50.8 in the control group, LS mean difference: − 3.2, 95% CI [− 9.57, 3.18], *p* = 0.3172) and in the PP analysis (LS mean difference: − 4.5, 95% CI [− 11.59, 0.86], *p* = 0.0890). Quality of life was comparable between the intervention and control groups in the mITT analysis regarding the physical subscale of the SF-12 (LS mean: 42.5 in the intervention vs. 42.1 in the control group, LS mean difference: 0.43, 95% CI [− 1.55, 2.4], *p* = 0.6493), and the mental subscale of the SF-12 (LS mean: 42.2 in the intervention vs. 43.4 in the control group, LS mean difference: − 1.2, 95% CI [− 7.11, 4.71], *p* = 0.6684). Results for the PP analysis for both subscales of the SF-12 were similar to the results of the mITT analysis (see Table 2).

## Discussion

To the best of our knowledge, there are no comparable randomized controlled trials in the literature on HCT in pediatric nephrology. The major aim of the study was to utilize improved medical care structures to boost adherence, with the long-term goal of improving transplant survival and thereby reducing morbidity and mortality.

In the intervention group, the BTP– which included specific consultation and counseling with a transition case manager – extended the standard of care for transition. However, we could not find any improvement in adherence or other outcome measures.

There was no statistically significant difference between the intervention and control groups, neither for primary nor for secondary endpoints.

Several reasons may explain these findings. As mentioned above, both groups began with a CoV associated with good medication adherence^14^, and it may be difficult to improve something, that is already highly satisfactory. This phenomenon might be due to a selection bias with adherent patients being more likely to participate in such trials than those who are more challenging and less adherent. In our previous retrospective census, the CoV was 20 ± 10%^14^. In the present trial, both groups started with an even better CoV which might indicate above-average motivation to comply. The prolonged recruiting time with comparably large numbers of patients unwilling to participate would support this theory.

Another analysis of the retrospective element of the TransNephro study showed that 76% of centers in Germany and Austria claimed to apply a transition procedure that was mutually agreed on even if it was unwritten^19^. So, the BTP may have been compared to many local but not necessarily inadequate, perhaps even structured, transition procedures. This may explain why the intervention and control groups in this trial barely differed.

The age at transfer in the present study was on average 19 years. Most guidelines agree that HCTshould be initiated years beforehand, sometimes as early as the age of 11 years^2,3^. Initiating the BTP only one year before transfer is likely far too late, as centers may have already prepared patients for transition years in advanceThis may be the reason why the BTP demonstrated no effect in our cohort.

We found only one comparable randomized controlled study on HCT but dealing with AYA with diabetes mellitus type 1. In the “Multicenter Canadian Randomized Controlled Trial of Structured Transition in Young Adults With Type 1 Diabetes”, published in 2019, a total of 104 young adults with structured transition were randomized against 101 who received “standard care”. The intervention lasted 18 months, 6 during pediatric and 12 during adult care. At the end of the 12 months under adult care no differences were found between either group with regard to HbA1c and clinic appointment adherence^8^.

SF-12 results were slightly below the general population norm^20^ yet al.so below the results of other research populations following KTx^21^. Our results on HRQoL are more consistent with studies on AYA with dialysis-dependent kidney-disease^22^. Unfortunately, our data do not provide any explanation for the HRQoL aspect and there was no difference between groups, implying that the additional support provided by the transition program did not affect this parameter. The low QOL (relative to previous studies) might have affected the ability of the patients to fully participate in and benefit from the intervention that was provided.

Study discontinuation was substantially higher in the intervention group. Some patients gave the reason for dropping out as a dislike of the case-management; however, it is not clear whether this was due to the concept or the call handler. This underlines the fact that the BTPmight work for many—but not all—adolescents.

Our study has several relevant limitations. It was underpowered to detect differences in rejection rates, graft loss rates, or eGFR because during study design it seemed feasible to recruit enough patients to use eGFR as a primary endpoint and medication adherence was therefore selected as an important surrogate parameter. However, even after recruiting for 52 months, we were unable to randomize the 114 patients calculated as necessary for acceptable statistical power concerning the primary endpoint. This highlights that either study centers and/or patients were not all convinced by the BTP approach, making recruitment difficult. Since we refrained from taking extra blood samples for the study, and left the parameters to the centers since adult nephrologists could not be expected to perform these, some useful values are missing, e.g., Cystatin C. The most relevant limitation is the very challenging documentation and data transfer due to the large number of patients lost to follow up after transfer combined with 10% of adult nephrologists refusing to cooperate with the study team despite agreement before the start of the study. In addition, we also received incomplete patient data from pediatric centers. The probability is that a stand-alone app, as used in this trial, is not sufficient on its own to promote behavioral change. In future trials, an integration of medication reminders and tracking by leveraging the most common apps currently used by children (e.g., Discord or Snapchat) could help to increase engagement.

For this multicenter trial of all German and Austrian pediatric KTx centers, it was not possible to look for electronically measured adherence as a secondary endpoint.

## Conclusion

The BTP as applied in our trial did not improve outcome in an adherent cohort of adolescents after KTx. Because of the problems we encountered in our trial, we think that it will be difficult to design and finance future multicenter HCT studies that include multiple interventions to improve transition and prevent the decrease in graft function.

## Electronic supplementary material

Below is the link to the electronic supplementary material.

Supplementary Material 1

## Data Availability

Clinical data will be provides by the authors upon request. Please contact: lars.pape@uk-essen.de.

## Abbreviations

ANCOVA: Analysis of covariance
AYA: Adolescents and young adults
BTP: Berlin Transition Program
CI: Confidence interval
CoV: Coefficient of variation
E: Concomitant entry
eCRF: Electronic case report form
eGFR: Estimated glomerular filtration rate
HCT: Health Care Transition
HRQoL: Health-related quality of life
ITT: Intention to treat
KTx: Kidney transplantation
LS: Least square
mITT: Modified intention-to-treat
PP: Per-protocol
QoL: Quality of life
T0: Time point 0/baseline
T1: Time point 12 (to 14)-month assessment
T2: Time point 24 (to 26)-month assessment
