# Supplementary material for: Results of a multicenter, randomized trial examining a new transition model for post-kidney transplant adolescents
Source: Sci Rep. 2025 Apr 3;15:11459. doi: 10.1038/s41598-025-95845-7 (PMC11968839; doi:10.1038/s41598-025-95845-7)
Supplement: Supplementary file 1 — Supplementary Material 1 [file 41598_2025_95845_MOESM1_ESM.pdf]

Supplementary Materials

The TRANSNephro-study examining a new transition model for post-kidney transplant adolescents - Results of a multicenter, randomized-controlled trial

Martin Kreuzer[1*], Jenny Prüfe[1*], Marie-Luise Dierks[2], Silvia Müther[3], Dirk Bethe[4], Anja Büscher[1], Krisztina Heindl-Rusai[5], Sabine Hollenbach[6], Bernd Hoppe[7], Ulrike John-Kroegel[8], Nele K Kanzelmeyer[9], Günter Klaus[10], Birgitta Kranz[11], Jun Oh[12], Martin Pohl[13], Susanne Rieger[4], Bettina Ruckenbrodt[14], Katja Sauerstein[15], Hagen Staude[16], Christina Taylan[17], Julia Thumfart[18], Marcus Weitz[19], Rieke Ringlstetter[20], Anika Großhennig[20*], Lars Pape[1*] and the Study group of the German Society for Pediatric Nephrology

Supplementary Methods

I. *Definition of **per protocol population***

In the PP population, we included all patients that fulfill the following criteria:

1) Immunosuppressive agent trough levels available for at least 8 months before transition
2) Immunosuppressive agent trough levels available for at least 8 months after transition
3) Transition was implemented
4) No graft loss
5) No dialysis

II. *Statistical Methods for secondary endpoints*

1) eGFR was estimated as proposed by Schwartz et al. (Schwartz GJ, Schneider MF, Maier PS, Moxey-Mims M, Dharnidharka VR, Warady BA, Furth SL, Munoz A):

   $$eGFR = 0.413 * \text{height (in cm)} / \text{Serum Creatinine (mg/dl)}$$

   We used the height at randomization for all estimations of eGFR during the course of the study.

2) SF-12 [16]: To be able to evaluate the SF-12 questionnaire one item of the questionnaire had to be recoded because of an incorrect survey of Item "SOC2" (*how*

often have your physical health or mental problem in the past four weeks affected your contacts with other people (visits to friends, relatives, etc.)?) The variable in the SF12 questionnaire has originally five possible answers: 1- all of the time, 2- most of the time, 3- some of the time, 4- a little of the time, and 5- none of the time. Unfortunately, the questionnaire used in the study had one answer option too many. Besides the five answer options described, the option “a good bit of a time” was also surveyed. Since a pre-defined code from the SF12 manual was used to evaluate the SF12 questionnaire, deviations in the variables were not possible. We recoded the variable so that all answers that were “most of the time” and “a good bit of a time” were summarized into “most of the time”.

Supplementary Results

Supplementary Table 1. Baseline characteristics of the ITT population

	**ITT**		
	Intervention	Control	p-Value
**n**	49	53	
**Gender, male (%)**	29 (59.2%)	27 (50.9%)	0.4034
**Age, years**	17.8 ± 1.7<br>(17 / 16-23)	17.6 ± 1.5<br>(17 / 15-22)	0.5117
**Height, cm**	166.1 ± 11.8<br>(168 / 130-185)	166.2 ± 10.0<br>(166 / 130-186)	0.9510
**Time since Tx, years**	7.6 ± 4.7<br>(7/ 0-15)	5.8 ± 4.7<br>(5/ 0-17)	0.0592
**Randomization stratum**<br>1 (male, time since Tx < 5 y)<br>2 (male, time since Tx ≥ 5 y)<br>3 (female, time since Tx < 5 y)<br>4 (female, time since Tx ≥ 5 y)	 10 (20.4%)<br>19 (38.8%)<br>9 (18.4%)<br>11 (22.5%)	 14 (26.4%)<br>13 (24.5%)<br>15 (28.3%)<br>11 (20.8%)	0.3706

Supplementary Table 2: Sensitivity analyses for primary and secondary endpoints

**Model**		**Population**	**LSMean**	**LSMean**	**p-value**
**Dependent**	**Adjustment**		**Intervention**	**Control**	
Variation coefficient of trough level	-	ITT (n = 81)	0.35	0.34	0.8327
	Randomization stratum	ITT (n = 81)	0.36	0.33	0.6470
	Randomization stratum, study site (according primary model)	ITT (n = 81)	0.31	0.29	0.8265
	-	PP (n = 60)	0.40	0.37	0.6998
	Randomization stratum	PP (n = 60)	0.39	0.36	0.6561
	Randomization stratum, study site (according primary model)	PP (n = 60)	0.32	0.32	0.9426
	-	ITT (n = 65)	45.37	52.66	0.1663

Mean eGFR at adult hospital phase	Randomization stratum	ITT (n = 65)	47.07	52.67	0.2713
	-	PP (n = 57)	48.4	55.9	0.1410
	Randomization stratum	PP (n = 57)	50.41	55.75	0.2537
SF-12 physical subscale at adult hospital phase	-	ITT (n = 35)	41.09	42.00	0.4996
	Randomization stratum	ITT (n = 35)	41.15	42.09	0.5013
	-	PP (n = 28)	41.84	41.63	0.8753
	Randomization stratum	PP (n = 28)	41.63	41.65	0.9907
SF-12 mental subscale at adult hospital phase	-	ITT (n = 35)	44.16	43.91	0.9057
	Randomization stratum	ITT (n = 35)	43.64	43.78	0.9482
	-	PP (n = 28)	44.57	42.98	0.5018
	Randomization stratum	PP (n = 28)	44.18	42.92	0.6217

Supplementary Figures

Boxplots of continuous outcome data during the course of the study

Supplementary Figure 1: Boxplots on CoV for mITT (A) and PP (B)

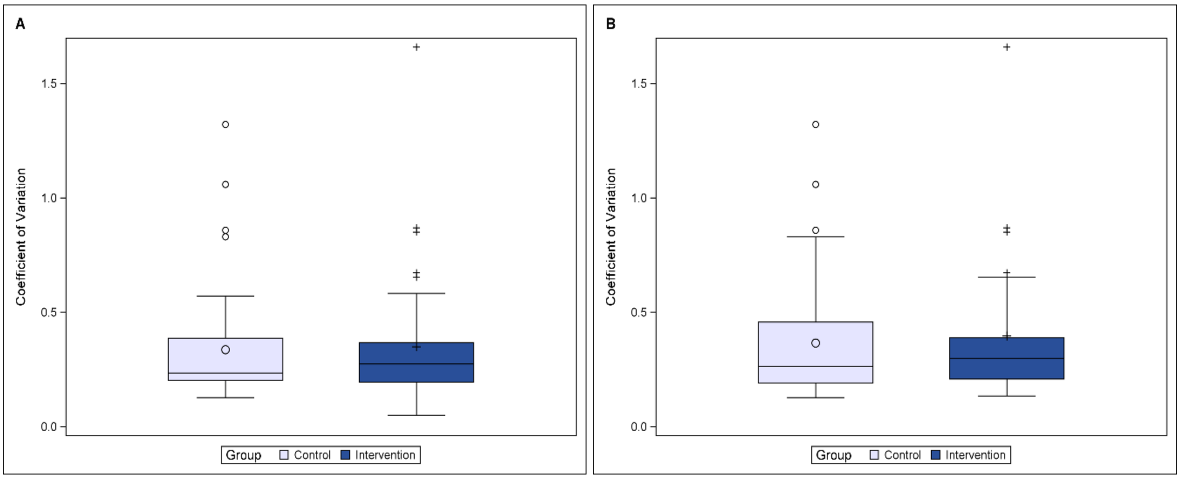

CoV – coefficient of variation of immuno-suppressant trough level

Supplementary Figure 2: Boxplots on mean eGFR for mITT (A) and PP (B)

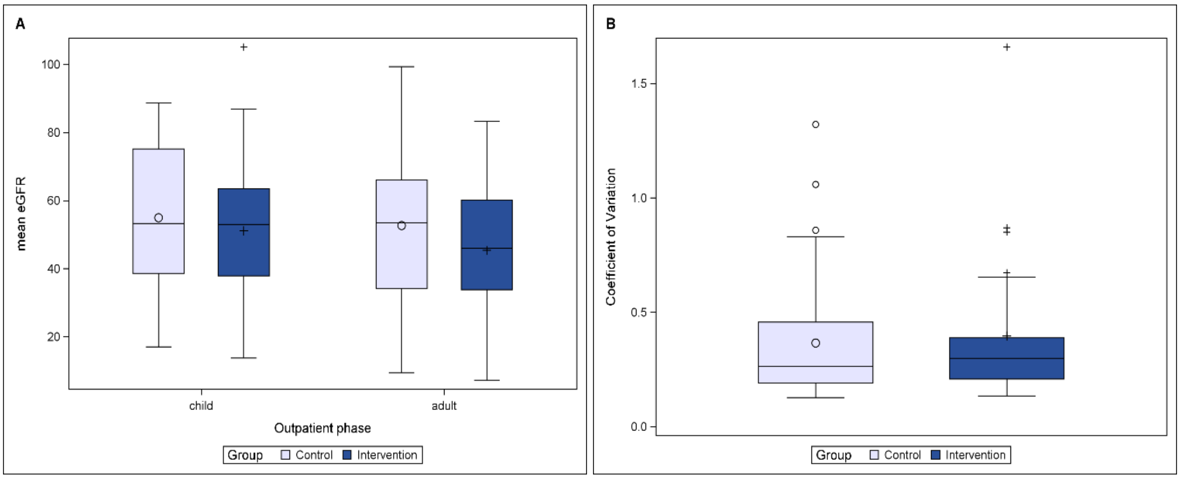

eGFR – estimated glomerular filtration rate

Supplementary Figure 2: Boxplots on SF-12 (all available data is depicted) for the physical scale (A) and the mental scale (B)

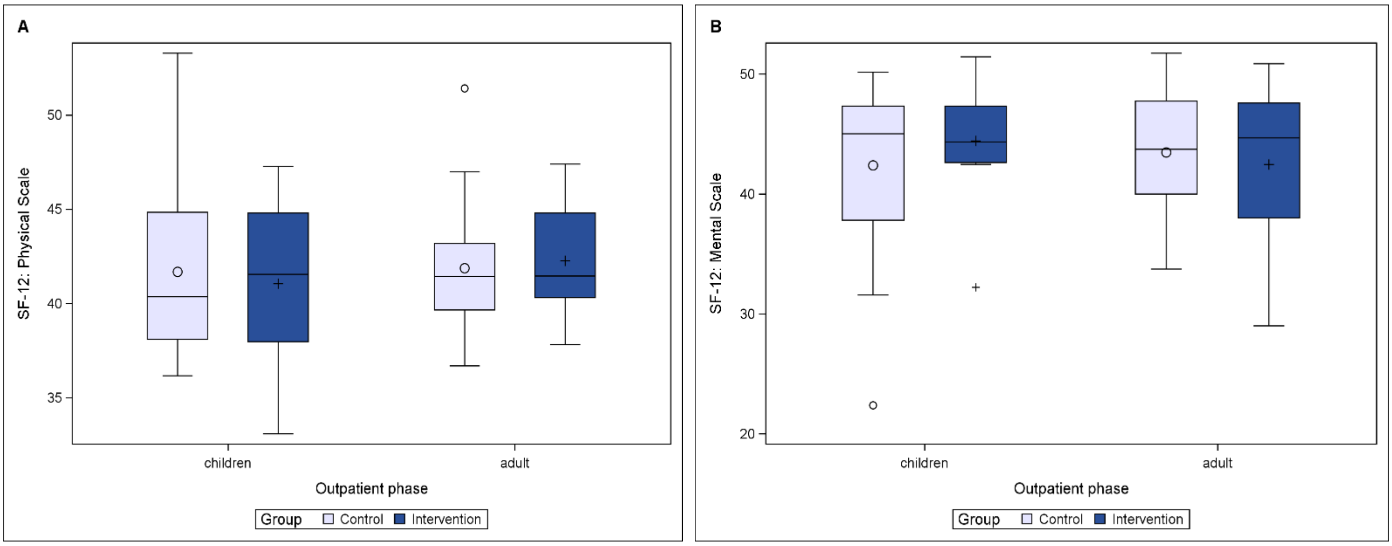

SF-12 – Short form-12 questionnaire
